# Post-load hyperglycemia as an important predictor of long-term adverse cardiac events after acute myocardial infarction: a scientific study

**DOI:** 10.1186/1475-2840-9-75

**Published:** 2010-11-11

**Authors:** Shuichi Kitada, Yoritaka Otsuka, Nobuaki Kokubu, Yoichiro Kasahara, Yu Kataoka, Teruo Noguchi, Yoichi Goto, Genjirou Kimura, Hiroshi Nonogi

**Affiliations:** 1 Department of Cardiology, National Cardiovascular Center, Osaka, Japan; 2 Department of Cardio-Renal Medicine and Hypertension, Nagoya City University Graduate School of Medical Sciences, Aichi, Japan; 3 Department of Cardiology, Fukuoka Wajiro Hospital, Fukuoka, Japan

## Abstract

**Background:**

Diabetes mellitus (DM) and impaired glucose tolerance (IGT) are risk factors for acute myocardial infarction (AMI). However, it is unknown whether hyperglycemic state is associated with increased major adverse cardiovascular events (MACE) after AMI. In this study, we evaluated the relationship between glucometabolic status and MACE in patients after AMI, and determined the critical level of 2 h post-load plasma glucose that may be used to predict MACE.

**Methods:**

AMI patients (n = 422) were divided into 4 groups as follows: normal glucose tolerance (NGT) group, IGT group, newly diagnosed DM (NDM) group, and previously known DM (PDM) group. MACE of the 4 groups were compared for 2 years from AMI onset.

**Results:**

The NDM group had a significantly higher event rate than the IGT and NGT groups and had a similar event rate curve to PDM group. The logistic models analyses revealed that 2 h post-load plasma glucose values of ≥160 mg/dL was the only independent predictor of long-term MACE after AMI (p = 0.028, OR: 1.85, 95% CI: 1.07-3.21). The 2-year cardiac event rate of patients with a 2 h post-load hyperglycemia of ≥160 mg/dL was significantly higher than that of patients with 2 h post-load glucose of <160 mg/dL (32.2% vs. 19.8%, p < 0.05) and was similar to that of PDM group (37.4%, p = 0.513).

**Conclusions:**

NDM increases the risk of MACE after AMI as does PDM. Particularly, post-AMI patients with a 2 h post-load hyperglycemia ≥160 mg/dL may need adjunctive therapy after AMI.

## Introduction

It is well known that diabetes mellitus (DM) is an independent risk factor for cardiovascular disease (CVD). The risk of CVD for diabetic patients is two to three times higher than that of subjects without DM [1-4]. In addition, several previous studies have shown that post-load hyperglycemia such as impaired glucose tolerance (IGT) is a risk factor for CVD [5-7]. In patients with acute myocardial infarction (AMI), the prevalence of abnormal glucose tolerance (AGT) using an oral glucose tolerance test (OGTT) was >70% [8-11]. In the GAMI (Glucose Abnormalities in Patients with Myocardial Infarction) study and the report by Tamita et al, AGT including IGT and newly diagnosed DM (NDM) were strong predictors of major adverse cardiac events (MACE) after AMI [12,13].

The risk for poor long-term prognosis after AMI may be already apparent if patients have post-load hyperglycemia even at plasma glucose level well below the diabetic threshold. The threshold for definition of DM is primarily based on a post-load two-hour (2 h) plasma glucose level in patients with diabetic retinopathy. Hence, cardiovascular disease has not been considered as a factor in the classification of glucose tolerance abnormality.

In the current study, we performed a retrospective analysis of patients with AMI whose glucose tolerance were assessed by using a 75 g OGTT, and evaluated the relationship between their glucometabolic status and long-term MACE. We also determined the critical level of the 2 h post-load plasma glucose to predict the occurrence of MACE.

## Methods

### Study Patients

Between January 2000 and December 2004, a total of 763 patients with first AMI were admitted to the National Cardiovascular Center (Osaka, Japan). Of the 763 patients admitted, 422 patients who received glucose tolerance tests or were previously known to have DM were evaluated for cardiac events for two years.

The diagnosis of AMI was based on the presence of typical chest pain, ST-segment change or new Q-waves in the 12-lead electrocardiogram, an increase of ≥2-fold over the upper limit of normal in creatine kinase concentration, local asynergy of left ventricular wall on ultrasound cardiography or left ventriculography, or occlusion of coronary artery on coronary angiography. Patients with transient left ventricular ballooning syndrome, vasospasm, spontaneous coronary artery dissection, thromboembolism, catheter related complications, left main trunk infarction, type 1 diabetes mellitus or familial hyperlipidemia were excluded from the study.

### Classification of abnormal glucose tolerance

Glucose abnormalities of study patients were classified into 4 groups using a 75 g OGTT according to the criteria for glucometabolic disturbances established by the World Health Organization (WHO). This was because we first started to evaluate abnormal glucose tolerance using a 75 g OGTT in patients after AMI in year 2000 [14,15]. Only 4 Patients with impaired fasting glucose tolerance were defined as IGT according to Japanese diabetes criteria. A 75 g OGTT was performed in the stable phase of AMI (median: 9 days from the onset of AMI). Previously known DM (PDM) patients were defined as those with clinical history of using oral hypoglycemic agents and/or insulin and patients with fasting plasma glucose levels ≥126 mg/dL. The other patients were urged to evaluate their glucose tolerance by a 75 g OGTT. NDM was defined as fasting glucose level <126 mg/dL and a 2 h post-load glucose level ≥200 mg/dL. Normal glucose tolerance (NGT) was defined as fasting glucose level <110 mg/dL and a 2 h post-load glucose level <140 mg/dL. In the remaining patients, IGT was defined as fasting glucose level <126 mg/dL and a 2 h post-load glucose level ≥140 mg/dL or with fasting glucose level of 110-125 mg/dL and a 2 h post-load glucose level <140 mg/dL.

### Data Collection

All patients who survived the acute phase (≤30 days) were followed-up at the outpatient clinic and their data for MACE were collected. Three patients with PDM died during the acute phase. In this study, a total of 422 patients were followed-up for two years. Patients' outcomes were assessed on the basis of MACE, which included death from cardiovascular causes, nonfatal AMI, hospitalization for heart failure and revascularization for restenosis and de novo lesions. Revascularization was performed after obtaining ischemic evidence by several modalities.

To determine the predictors of long-term MACE, the baseline clinical characteristics of the patients including laboratory data, characteristics of AMI, and medications (Table 1) were evaluated using hospital records under the auspices of the institutional cardiovascular outcomes monitoring program.

**Table 1:** Clinical Characteristics of Study Patients

	NGT	IGT	NDM	PDM
	(n = 106)	(n = 140)	(n = 68)	(n = 108)
**Basic characteristics**				
Male, n (%)	77 (72.6)	108 (77.1)	56 (82.4)	86 (79.6)
Age, year	64 ± 11	66 ± 10	63 ± 11	68 ± 9.* ***
Body-mass index, kg/m^2^	23.4 ± 3.0	23.5 ± 2.6	24.5 ± 2.7* **	24.2 ± 2.9* **
Family history of IHD, n (%)	30 (28.3)	45(32.1)	19 (27.9)	26 (24.1)
History of smoking, n (%)	80 (75.5)	99(70.7)	56 (82.4)	79 (73.1)
Clinical history, n (%)				
Hypertension, n (%)	66 (62.3)	82 (58.6)	41 (60.3)	63 (58.3)
Hyperlipidemia, n (%)	62 (58.5)	84 (60.0)	44 (64.7)	62 (57.4)
Heart failure, n (%)	2 (1.9)	2 (1.4)	0 (0.0)	2 (1.9)
Cerebrovascular disease, n (%)	4 (3.8)	12(8.6)	2 (2.9)	13 (12.0)*
Chronic renal failure, n (%)	2 (1.9)	9 (6.4)	1(1.5)	9 (8.3)
ASO, n (%)	5(4.7)	6 (4.3)	4 (5.9)	17 (15.7)* **
Chronic obstructive pulmonary disease, n (%)	2 (1.9)	5 (3.6)	1 (1.5)	1 (0.9)
Malignancy, n (%)	4 (3.8)	11 (7.9)	3 (4.4)	3 (2.8)
Previous procedures (PTCA/CABG), n (%)	3 (2.8)	7 (5.0)	6 (8.8)	8 (7.4)
**Laboratory data at the time of admission**				
Fasting blood sugar, mg/dL	90 ± 7	94 ± 9.*	99 ± 11* **	
Blood sugar after 2 h, mg/dL	115 ± 18	165 ± 20*	237 ± 30* **	
HbA1c, %	5.3 ± 0.3	5.4 ± 0.5*	5.7 ± 0.4* **	8.1 ± 1.5* ** ***
Total Cholesterol, mg/dL	194 ± 39	193 ± 37	199 ± 33	199 ± 42
Triglyceride, mg/dL	107 ± 85	102 ± 56	123 ± 74**	122 ± 87**
LDL-Cholesterol, mg/dL	130 ± 37	130 ± 32	129 ± 33	130 ± 36
HDL-Cholesterol, mg/dL	44 ± 11	43 ± 12	45 ± 20	45 ± 12
Serum creatinine, mg/dL	0.8 ± 0.2	0.8 ± 0.3	0.8 ± 0.2	0.9 ± 0.3
Urea acid, mg/dL	5.3 ± 1.5	5.5 ± 1.6	5.6 ± 1.3	5.2 ± 1.8
**Characteristics of acute myocardial infarction**				
Systolic blood pressure on admission, mmHg	135 ± 25	139 ± 23	139 ± 24	136 ± 24
Heart rate on admission, bpm/m	72 ± 17	75 ± 16	76 ± 18	77 ± 19
Killip class≧Ⅱ, n (%)	9 (8.5)	5 (3.6)	4 (5.9)	15 (13.9)**
Anterior MI, n (%)	45 (42.5)	77 (55.0)	28 (41.2)	50 (46.3)
Multi-vessel disease, n (%)	48(45.3)	63 (45.0)	35 (51.5)	73 (67.6)* ** ***
Procedural features				
Thrombolysis, n (%)	16 (15.1)	19 (13.6)	7 (10.3)	14 (13.0)
Primary PTCA/CABG, n (%)	64(60.4)	96 (68.6)	45 (66.2)	61 (56.5)
Stent implantation, n (%)	73(68.9)	116 (82.9)*	52 (76.5)	68 (63.0)
LVEF, %	44.4 ± 8.1	42.8 ± 8.2	43.9 ± 8.8	43.6 ± 9.2
Peak CPK, U/L	2827 ± 1905	2988 ± 2385	2741 ± 1588	2757 ± 2393
**Medications**				
Aspirin, n (%)	103(97.2)	138 (98.6)	66 (97.1)	104 (96.3)
ACEI/ARB, n (%)	72 (67.9)	104 (74.3)	54 (79.4)	73 (67.6)
Beta-blockers, n (%)	46(43.4)	87(62.1)*	45 (66.2)*	63(58.3)*
Statin, n (%)	46 (43.4)	65 (46.4)	39 (57.4)	45 (41.2)
Oral hypoglycemic agents, n (%)	0 (0)	0 (0)	2 (2.9)	66 (61.1)* ** ***
Insulin therapy, n (%)	0 (0)	0 (0)	0 (0)	18 (16.7)* ** ***

*; vs NGT p < 0.05 **; vs IGT p < 0.05 ***; vs NDM p < 0.05

NGT, normal glucose tolerance; IGT, impaired glucose tolerance; NDM, newly diagnosed diabetes mellitus; PDM, previously known diabetes mellitus; IHD, ischemic heart disease; ASO, arteriosclerosis obliterans; PTCA, percutaneous transluminal coronary angioplasty; CABG, coronary artery bypass grafting; HbA1c, hemoglobin A1c; LDL-Cholesterol, low-density lipoprotein cholesterol; HDL-Cholesterol, high-density lipoprotein Cholesterol; MI = myocardial infarction; LVEF, left ventricular ejection fraction; CPK, creatine phosphokinase; ACEI, angiotensin-converting enzyme inhibitor; ARB, angiotensin II receptor blocker

### Statistical Analyses

SPSS Software version 17.0 (Chicago, USA) was used for all statistical analyses. Continuous variables were expressed as mean ± standard deviation (SD). Statistical significance was evaluated using unpaired Student's *t *test for comparisons between two means, and a chi-square test for categorical data. Event-free survival curves were constructed using the Kaplan-Meier survival methods and were compared with log-rank statistics. Survival time was defined as the intervals between the onset of AMI and the time of MACE. Receiver-operating characteristic (ROC) analysis was performed to define sensitivity and specificity of 2 h post-load plasma glucose. In addition, ROC analysis was used as an exploratory evaluation of the best cut-off point of 2 h post-load plasma glucose to predict MACE after AMI in non-PDM patients and positive and negative predictive values were derived using this cut-off value. Logistic regression analysis was used to identify predictors of cardiac events. A *p *value of < 0.05 was considered statistically significant.

## Results

### Clinical backgrounds and prevalence of glucose abnormalities

**Figure 1:**
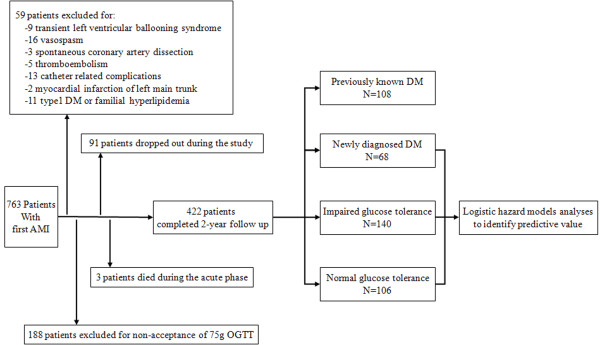
**Study profile**.

Figure 1 shows a flow diagram for the enrollment of patients into this study. Of the total 763 patients, we excluded a total of 247 patients; 48 patients for etiology of AMI, 11 patients for clinical history of type1 DM and familial hyperlipidemia, and 188 patients who did not consent to have the 75 g OGTT. Three patients with PDM died during the acute phase (≤30 days) after AMI. In all, 422 patients completed two years of follow-up from onset; 91 patients dropped out during the study and did not complete the two years of follow up. The clinical characteristics of the patients who completed the study are shown in Table 1. The PDM group had a higher mean age, and a higher prevalence of co-morbidities and incidence of multi-vessel disease compared with the other groups. The NDM and PDM groups had higher body-mass index and serum triglyceride levels compared with the NGT and IGT groups. The level of HbA1c of the NDM group was significantly lower than that of the PDM group (5.7 ± 0.4 mg/dL vs. 8.1 ± 1.5 mg/dL, p <0.05). Only 2.9% of patients in the NDM group were being treated by oral hypoglycemic agents, while 61.1% and 16.7% of patients in the PDM group were being treated by oral hypoglycemic agents and insulin therapy, respectively. The prevalence of NGT, IGT, NDM, and PDM was 25%, 32%, 16%, and 26%, respectively.

Of the 310 patients who were tested with 75 g OGTT, 204 patients (66%) showed post-load hyperglycemia of ≥140 mg/dL. Only 4 patients showed impaired fasting glucose tolerance with a fasting blood glucose level of 110 mg/dL to 126 mg/dL.

### Long-term MACE

**Figure 2:**
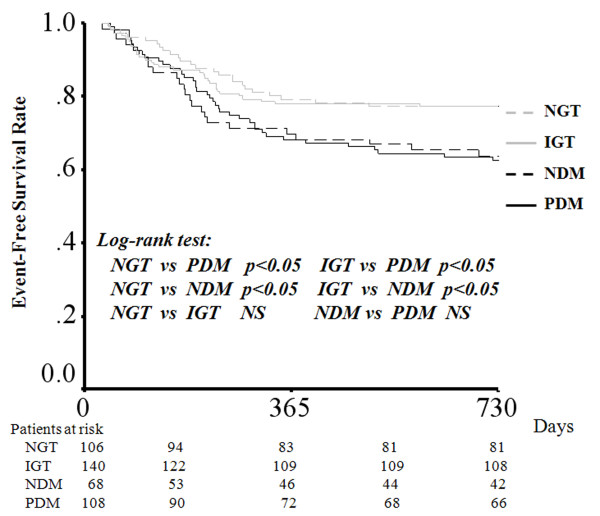
**Kaplan-Meier curves showing event-free survival from MACE after AMI during the 2-year follow-up among the 4 groups**.

Long-term MACE were observed in 120 patients. Table 2 shows the numbers of long-term MACE among study patients. There were 40 cardiovascular events in the PDM group, 24 events in the NDM group, 32 events in the IGT group, and 24 events in the NGT group. The 2-year cumulative event free rates from MACE were 63.0% for the PDM group, 64.7% for the NDM group, 77.1% for the IGT group, and 77.4% for the NGT group. Figure 2 shows Kaplan-Meier plots of study patients with MACE during the 2-year follow-up period. The PDM group and the NDM group showed significantly higher event rates than non-diabetic patients and the NDM group had a similar event rate to the PDM group. There was no significant difference in event free rate between the IGT group and the NGT group during the follow-up period.

**Table 2:** Long-term MACE in Patients with Acute Myocardial Infarction during 2-year Follow-up

	Long-term MACE (>30 days)			
	
	NGT	IGT	NDM	PDM
	
	106	140	68	108
Cardiac death, n (%)	0	0	0	4 (4)
Non-fatal acute myocardial infarction, n (%)	0	2 (1)	2 (3)	1 (1)
Hospitalization for heart failure, n (%)	3 (3)	3 (2)	1 (1)	4 (4)
Revascularization				
Target vessel revascularization, n (%)	18 (17)	20 (14)	19 (28)	25 (23)
Revascularization to de novo lesion, n (%)	3 (3)	7 (5)	2 (3)	6 (6)

Total MACE, n (%)	24 (23)	32 (23)	24 (35)	40 (37)

MACE, major adverse cardiovascular events; NGT, normal glucose tolerance; IGT, impaired glucose tolerance; NDM, newly diagnosed diabetes mellitus; PDM, previously known diabetes mellitus

### Predictors of long-term MACE

The clinical characteristics were compared between non-PDM patients with and without MACE (Table 3). There was no significant difference in long-term MACE rate between non-PDM patients with and without MACE although the 2 h post-load plasma glucose appeared to be related to the occurrence of MACE. Logistic analyses were carried out to examine an independent predictive value of the 2 h post-load plasma glucose level for long-term MACE. The adjusted variables were the 5 factors that were previously reported to be related to MACE: age, left ventricular ejection fraction, multi-vessel disease, use of beta-blockers, and statin use.

**Table 3:** Comparison of Clinical Characteristics between Patients with and without Adverse Cardiovascular Events not Previously Known to have Diabetes Mellitus

	**Patientswith Cardiovascular Events**	Patients without Cardiovascular Events	
	(n = 80 )	(n = 234)	p value
**Basic characteristics**			
Male, n (%)	59 (73.8)	182 (78.1)	0.44
Age, year	64 ± 11	64 ± 10	0.91
Body-mass index, kg/m^2^	23.5 ± 2.8	23.7 ± 2.8	0.71
Family history of IHD, n (%)	26 (32.5)	68 (29.2)	0.58
History of smoking, n (%)	58(72.5)	177 (76.0)	0.55
Clinical history			
Hypertension, n (%)	45 (56.3)	144 (61.8)	0.43
Hyperlipidemia, n (%)	52 (65.0)	137 (58.8)	0.36
Heart failure, n (%)	1 (1.3)	3 (1.3)	>0.99
Cerebrovascular disease, n (%)	4 (5.0)	14 (6.0)	>0.99
Chronic renal failure, n (%)	2 (2.5)	10 (4.3)	0.74
ASO, n (%)	6 (7.5)	9 (3.9)	0.22
Chronic obstructive pulmonary disease, n (%)	1 (1.3)	7 (3.0)	0.69
Malignancy, n (%)	5 (6.3)	12 (5.2)	0.78
Previous procedures (PTCA/CABG), n (%)	6 (7.5)	10 (4.3)	0.25
**Laboratory data at the time of admission**			
Fasting blood sugar, mg/dL	95 ± 9	94 ± 10	0.45
Blood sugar after 2 h, mg/dL	172 ± 51	160 ± 49	0.07
HbA1c, %	5.4 ± 0.5	5.4 ± 0.4	0.75
Total Cholesterol, mg/dL	198 ± 34	194 ± 38	0.34
Triglyceride, mg/dL	109 ± 73	107 ± 71	0.85
LDL-Cholesterol, mg/dL	134 ± 34	128 ± 34	0.18
HDL-Cholesterol, mg/dL	42 ± 9	44 ± 15	0.16
Serum creatinine, mg/dL	0.8 ± 0.2	0.8 ± 0.3	0.66
Urea acid, mg/dL	5.4 ± 1.6	5.5 ± 1.5	0.75
**Characteristics of acute myocardial infarction**			
Systolic blood pressure on admission, mmHg	136 ± 26	138 ± 23	0.65
Heart rate on admission, bpm/m	77 ± 18	73 ± 16	0.14
Killip class≧Ⅱ, n (%)	7 (8.8)	11 (4.7)	0.26
Anterior MI, n (%)	42 (52.5)	108 (46.4)	0.37
Multi-vessel disease, n (%)	42 (52.5)	104 (44.6)	0.24
Procedural features			
Thrombolysis, n (%)	12 (15.0)	30 (12.9)	0.7
Primary PTCA/CABG, n (%)	47 (58.8)	158 (67.8)	0.17
Stent implantation, n (%)	63 (78.8)	178 (76.4)	0.75
LVEF, %	42.2 ± 8.5	44.0 ± 8.2	0.11
Peak CPK, U/L	2965 ± 1836	2857 ± 2154	0.71
**Medications**			
Aspirin, n (%)	79 (98.8)	227 (97.4)	0.68
ACEI/ARB, n (%)	59 (73.8)	171 (73.4)	>0.99
Beta-blockers, n (%)	53 (66.3)	125 (53.6)	0.07
Statin, n (%)	37 (46.3)	112 (48.1)	0.8
Oral hypoglycemic agents, n (%)	1 (1.3)	1 (0.4)	0.45

IHD, ischemic heart disease; ASO, arteriosclerosis obliterans; PTCA, percutaneous transluminal coronary angioplasty; CABG, coronary artery bypass grafting; HbA1c, hemoglobin A1c; LDL-Cholesterol, low-density lipoprotein cholesterol; HDL-Cholesterol, high-density lipoprotein Cholesterol; MI, myocardial infarction; LVEF, left ventricular ejection fraction; CPK, creatine phosphokinase; ACEI, angiotensin-converting enzyme inhibitor; ARB, angiotensin II receptor blocker

**Figure 3:**
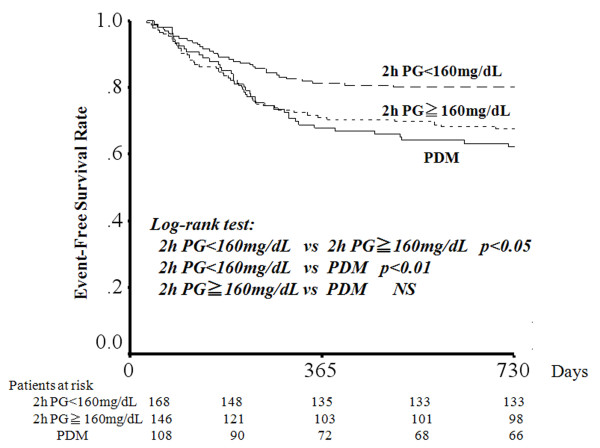
**Kaplan-Meier curves showing event-free survival from MACE after AMI among patients with 2 h post-load plasma glucose ≥160 mg/dL, with 2 h post-load plasma glucose <160 mg/dL, and with previously known DM**.

The ROC analysis indicated that a cut-off value of 2 h post-load plasma glucose level ≥160 mg/dL best predicted MACE in non-PDM patients. This measure of post-load glucose value showed a sensitivity and specificity of 59% and 58%, respectively. The positive and negative predictive values of 2 h post-load plasma glucose level ≥160 mg/dL were 32% and 80%, respectively. The odds ratio of 2 h post-load plasma glucose values for MACE was 1.85 (95% CI: 1.07-3.21) for patients with glucose level ≥160 mg/dL. The logistic models analyses revealed that 2 h post-load plasma glucose values of ≥160 mg/dL was the only independent predictor of long-term MACE after AMI (p = 0.028, OR: 1.85, 95% CI: 1.07-3.21). Event-free survival rate was also compared between patients with 2 h post-load plasma glucose ≥160 mg/dL vs. <160 mg/dL, and patients with 2 h post-load plasma glucose ≥160 mg/dL vs. PDM group. The 2-year event-free rate of patients with 2 h post-load plasma glucose ≥160 mg/dL was significantly higher than that of patients with post-load plasma glucose <160 mg/dL (67.8% vs. 80.2%, p <0.05) and was similar to that of PDM group (62.6%, p = 0.513) (Figure 3).

## Discussion

### Relationship between glucometabolic status and long-term MACE

During the 2-year follow-up, the incidence of cardiovascular events after AMI was 37.0% in patients with PDM and 35.3% in patients with NDM. Both groups had similarly increased risk of long-term MACE after AMI and significantly higher incidence of MACE compared with non-diabetic patients including IGT patients. When baseline characteristics were compared between the NDM and PDM groups, the level of HbA1c of NDM group was significantly lower than that of PDM group (5.7 ± 0.4 mg/dL vs. 8.1 ± 1.5 mg/dL, p <0.05). These results indicate that patients with diabetes may have an increased risk of cardiovascular events after AMI irrespective of diabetic status such as severity or disease duration of DM. Therefore, early detection of NDM using a 75 g OGTT after AMI may be important in the risk assessment of cardiac events.

Several previous epidemiological studies have indicated that even patients with pre-diabetic conditions, below the diabetic threshold, are at high risk of cardiovascular disease [5-7]. The GAMI study and a study by Tamita et al [13] showed that AGT including IGT and NDM was a risk factor of increased cardiac events after AMI [12,13]. However, it is unclear whether patients with post-load hyperglycemia below the threshold for DM after AMI have a risk of long-term cardiovascular events. The Euro Heart Survey has reported that PDM or NDM, but not IGT, has a negative influence on the 1-year outcome in patients with coronary artery disease [16]. In the current study, we similarly demonstrated that NDM is a factor for increased risk of cardiac events as is PDM after AMI, but there was no significant difference for long-term MACE between IGT and NGT patients.

### Predictive value of 2 h post-load plasma glucose for long-term MACE

We demonstrated that patients with a 2 h post-load plasma glucose value ≥160 mg/dL, including patients with plasma glucose value consistent with DM, had a significantly higher risk of long-term MACE after AMI than the patients with post-load glucose value <160 mg/dL. Although we demonstrated NDM patients had an increased risk of cardiac events after AMI in the present study, we confirmed that patients with post-load hyperglycemia (≥160 mg/dL) had increased risk of poor long-term outcomes after AMI. Previous investigators have suggested several potential predictors for long-term cardiovascular events in patients with AMI, including impaired left ventricular function, residual ischemia, and pharmacological therapy [17-25]. Even after adjustment for previously proposed predictors and age, we found that a 2 h post-load plasma glucose level ≥160 mg/dL is another potential predictor of long-term cardiovascular events after AMI. The cut-off points for DM on fasting and 2 h post-load glucose values were primarily determined based on the prevalence of microvascular disease related to hyperglycemic complications like diabetic retinopathy. We may need to reconsider the thresholds used to diagnose post-load hyperglycemia for macrovascular disease or cardiac events after AMI.

### Relationship between long-term MACE and post-load hyperglycemia

Previous studies have shown that elevated plasma glucose adversely affects endothelium-dependent vasodilation, inflammatory responses, and increases oxidative stress on the cardiovascular system [26-30]. These hyperglycemic stresses may appear below the threshold glucose level for DM and play an important role for promoting cardiovascular events after AMI. Fasting glucose and glycated haemoglobin provide no information on glucose metabolism after glucose load. It is important to use an OGTT to evaluate glucose tolerance among patients with AMI [31,32]. Because we demonstrated that patients with a glucose value ≥160 mg/dL had a high risk of long-term MACE, similar to the PDM group, early detection of post-load hyperglycemia of ≥160 mg/dL using an OGTT may be useful in the risk management of patients with AMI. In addition, adjunctive therapy for these patients may improve long-term cardiac events after AMI. Further study is needed to confirm this observation.

### Usefulness of OGTT for patients with AMI

In the current study, 74% of the study patients showed abnormal glucose tolerance (32% for IGT, 16% for NDM, 26% for PDM). Recent studies have shown that NDM and IGT are common among patients with coronary artery disease including acute coronary syndrome [8-11,33,34]. According to the European registry, when patients with AMI, but without PDM, were challenged with an OGTT, about 65% showed abnormal glucose regulation [33]. We found that 74% of study patients had abnormal glucose tolerance, which was similar to the 65% reported previously [33].

### Clinical perspectives of intervention for post-load hyperglycemia

Although several previous studies have confirmed that post-load hyperglycemia or impaired fasting glucose increases cardiovascular disease morbidity and mortality [35-37], it is still unknown whether lifestyle modification and medication for hyperglycemia would reduce this risk. However, in the report from the Euro Heart Survey on Diabetes and the Heart [38], it has been noted that lowering blood glucose using metformin may reduce the risk of cardiovascular events among patients with DM. According to the PROACTIVE Study [39], pioglitazone significantly reduced the occurrence of cardiovascular events in patients with DM who have a high risk of macrovascular events. Recently, preliminary data have shown that there was a pronounced decrease in cardiovascular events in patients with NDM prescribed glucose-lowering drugs compared with those not receiving such treatment [38]. On the other hand, it has not been ascertained that for patients with IGT such interventions also reduce the risk of cardiovascular events. The STOP-NIDDM study [40] showed that the rate of cardiovascular events was significantly reduced in the patients with IGT who received acarbose compared with placebo. In a further study, we have to address the issue of whether the control of post-load hyperglycemia would reduce the risk of recurrence of cardiovascular events after AMI.

### Study limitations

This study was a nonrandomized retrospective analysis based on a small number of patients in a single center. Therefore, our results may not reflect the real world population. Second, we excluded from the study 188 patients (25%) who did not consent to have the 75 g OGTT. Enrolling more patients who consent to having the 75 g OGTT might make the results more convincing. In this study, we evaluated glucose tolerance of study patients at only two time points, fasting and 2 h post-load, using a 75 g OGTT. Estimating of post-load hyperglycemia and parameters such as plasma insulin level could better clarify the relationship of post-load hyperglycemia and cardiovascular events after AMI [41]. Large randomized prospective clinical trials are needed to support our conclusions.

## Conclusions

NDM increases the risk of MACE after AMI as does PDM. NDM and PDM patients have a similar poor prognosis for MACE after AMI. Particularly, post-AMI patients with a 2 h post-load hyperglycemia ≥160 mg/dL may need adjunctive therapy after AMI.

## Abbreviations

DM: diabetes mellitus; CVD: cardiovascular disease; IGT: impaired glucose tolerance; AMI: is acute myocardial infarction; AGT: abnormal glucose tolerance; OGTT: oral glucose tolerance test; WHO: World Health Organization; PDM: previously known DM; NDM: newly diagnosed DM; NGT: normal glucose tolerance; MACE: major adverse cardiac events.

## Competing interests

The authors declare that they have no competing interests.

## Authors' contributions

SK has made substantial contributions to acquisition, analysis and interpretation of data, and has been involved in drafting the manuscript. YO has made substantial contributions to conception and design and has been involved in revising the manuscript critically for important intellectual content. NK, YK, YK, TN, YG, GK and HN have been involved in revising the manuscript critically for important intellectual content. All authors have given final approval of the version to be published.

## Authors' information

SK: Interventional Cardiologist

YO: Interventional Cardiologist, FACC, FESC, and Director of catheterization laboratory

NK: Interventional Cardiologist

YK: Interventional Cardiologist

YK: Interventional Cardiologist

TN: General Cardiologist

YG: Director of Cardiology

GK: Professor of Nagoya City University Graduate School of Medical Sciences

HN: Director of Cardiology

## References

[B1] Stamler J Vaccaro O Neaton JD Wentworth D Diabetes, other risk factors, and 12-yr cardiovascular mortality for men screened in the Multiple Risk Factor Intervention Trial Diabetes Care 1993 16 434 444 10.2337/diacare.16.2.434 8432214

[B2] Gerstein HC Yusuf S Dysglycaemia and risk of cardiovascular disease Lancet 1996 347 949 950 10.1016/S0140-6736(96)91420-8 8598762

[B3] Schernthaner G Cardiovascular mortality and morbidity in type-2 diabetes mellitus Diabetes Res Clin Pract 1996 31 Suppl S3 13 10.1016/0168-8227(96)01224-7 8864635

[B4] Laakso M Hyperglycemia and cardiovascular disease in type 2 diabetes Diabetes 1999 48 937 942 10.2337/diabetes.48.5.937 10331395

[B5] Fujishima M Kiyohara Y Kato I Ohmura T Iwamoto H Nakayama K Ohmori S Yoshitake T Diabetes and cardiovascular disease in a prospective population survey in Japan: The Hisayama Study Diabetes 1996 45 Suppl 3 S14 16 8674881 10.2337/diab.45.3.s14

[B6] Tominaga M Eguchi H Manaka H Igarashi K Kato T Sekikawa A Impaired glucose tolerance is a risk factor for cardiovascular disease, but not impaired fasting glucose. The Funagata Diabetes Study Diabetes Care 1999 22 920 924 10.2337/diacare.22.6.920 10372242

[B7] Glucose tolerance and mortality: comparison of WHO and American Diabetes Association diagnostic criteria. The DECODE study group. European Diabetes Epidemiology Group. Diabetes Epidemiology Collaborative analysis Of Diagnostic criteria in Europe Lancet 1999 354 617 621 10.1016/S0140-6736(98)12131-1 10466661

[B8] Bartnik M Malmberg K Hamsten A Efendic S Norhammar A Silveira A Tenerz A Ohrvik J Ryden L Abnormal glucose tolerance--a common risk factor in patients with acute myocardial infarction in comparison with population-based controls J Intern Med 2004 256 288 297 10.1111/j.1365-2796.2004.01371.x 15367171

[B9] Hashimoto K Ikewaki K Yagi H Nagasawa H Imamoto S Shibata T Mochizuki S Glucose intolerance is common in Japanese patients with acute coronary syndrome who were not previously diagnosed with diabetes Diabetes Care 2005 28 1182 1186 10.2337/diacare.28.5.1182 15855586

[B10] Ramachandran A Chamukuttan S Immaneni S Shanmugam RM Vishnu N Viswanathan V Jaakko T High incidence of glucose intolerance in Asian-Indian subjects with acute coronary syndrome Diabetes Care 2005 28 2492 2496 10.2337/diacare.28.10.2492 16186285

[B11] Norhammar A Tenerz A Nilsson G Hamsten A Efendic S Ryden L Malmberg K Glucose metabolism in patients with acute myocardial infarction and no previous diagnosis of diabetes mellitus: a prospective study Lancet 2002 359 2140 2144 10.1016/S0140-6736(02)09089-X 12090978

[B12] Bartnik M Malmberg K Norhammar A Tenerz A Ohrvik J Ryden L Newly detected abnormal glucose tolerance: an important predictor of long-term outcome after myocardial infarction Eur Heart J 2004 25 1990 1997 10.1016/j.ehj.2004.09.021 15541834

[B13] Tamita K Katayama M Takagi T Akasaka T Yamamuro A Kaji S Morioka S Kihara Y Impact of newly diagnosed abnormal glucose tolerance on long-term prognosis in patients with acute myocardial infarction Circ J 2007 71 834 841 10.1253/circj.71.834 17526977

[B14] Report of the Expert Committee on the Diagnosis and Classification of Diabetes Mellitus Diabetes Care 1997 20 1183 1197 9203460 10.2337/diacare.20.7.1183

[B15] Alberti KG Zimmet PZ Definition, diagnosis and classification of diabetes mellitus and its complications. Part 1: diagnosis and classification of diabetes mellitus provisional report of a WHO consultation Diabet Med 1998 15 539 553 10.1002/(SICI)1096-9136(199807)15:7<539::AID-DIA668>3.0.CO;2-S 9686693

[B16] Lenzen M Ryden L Ohrvik J Bartnik M Malmberg K Scholte Op Reimer W Simoons ML Diabetes known or newly detected, but not impaired glucose regulation, has a negative influence on 1-year outcome in patients with coronary artery disease: a report from the Euro Heart Survey on diabetes and the heart Eur Heart J 2006 27 2969 2974 10.1093/eurheartj/ehl363 17090612

[B17] Collaborative meta-analysis of randomised trials of antiplatelet therapy for prevention of death, myocardial infarction, and stroke in high risk patients BMJ 2002 324 71 86 10.1136/bmj.324.7329.71 11786451 PMC64503

[B18] Peters RJ Mehta SR Fox KA Zhao F Lewis BS Kopecky SL Diaz R Commerford PJ Valentin V Yusuf S Effects of aspirin dose when used alone or in combination with clopidogrel in patients with acute coronary syndromes: observations from the Clopidogrel in Unstable angina to prevent Recurrent Events (CURE) study Circulation 2003 108 1682 1687 10.1161/01.CIR.0000091201.39590.CB 14504182

[B19] Metoprolol in acute myocardial infarction (MIAMI) A randomised placebo-controlled international trial. The MIAMI Trial Research Group Eur Heart J 1985 6 199 226 2863148

[B20] Randomised trial of intravenous atenolol among 16 027 cases of suspected acute myocardial infarction: ISIS-1. First International Study of Infarct Survival Collaborative Group Lancet 1986 2 57 66 10.1016/S0140-6736(02)92899-0 2873379

[B21] Yusuf S Sleight P Pogue J Bosch J Davies R Dagenais G Effects of an angiotensin-converting-enzyme inhibitor, ramipril, on cardiovascular events in high-risk patients. The Heart Outcomes Prevention Evaluation Study Investigators N Engl J Med 2000 342 145 153 10.1056/NEJM200001203420301 10639539

[B22] Fox KM Efficacy of perindopril in reduction of cardiovascular events among patients with stable coronary artery disease: randomised, double-blind, placebo-controlled, multicentre trial (the EUROPA study) Lancet 2003 362 782 788 10.1016/S0140-6736(03)14974-4 13678872

[B23] Randomised trial of cholesterol lowering in 4444 patients with coronary heart disease: the Scandinavian Simvastatin Survival Study (4S) Lancet 1994 344 1383 1389 7968073

[B24] Sacks FM Pfeffer MA Moye LA Rouleau JL Rutherford JD Cole TG Brown L Warnica JW Arnold JM Wun CC Davis BR Braunwald E The effect of pravastatin on coronary events after myocardial infarction in patients with average cholesterol levels. Cholesterol and Recurrent Events Trial investigators N Engl J Med 1996 335 1001 1009 10.1056/NEJM199610033351401 8801446

[B25] Prevention of cardiovascular events and death with pravastatin in patients with coronary heart disease and a broad range of initial cholesterol levels. The Long-Term Intervention with Pravastatin in Ischaemic Disease (LIPID) Study Group N Engl J Med 1998 339 1349 1357 10.1056/NEJM199811053391902 9841303

[B26] Ceriello A The post-prandial state and cardiovascular disease: relevance to diabetes mellitus Diabetes Metab Res Rev 2000 16 125 132 10.1002/(SICI)1520-7560(200003/04)16:2<125::AID-DMRR90>3.0.CO;2-4 10751752

[B27] De Vriese AS Verbeuren TJ Van de Voorde J Lameire NH Vanhoutte PM Endothelial dysfunction in diabetes Br J Pharmacol 2000 130 963 974 10.1038/sj.bjp.0703393 10882379 PMC1572156

[B28] Temelkova-Kurktschiev T Henkel E Koehler C Karrei K Hanefeld M Subclinical inflammation in newly detected Type II diabetes and impaired glucose tolerance Diabetologia 2002 45 151 10.1007/s125-002-8256-1 11845235

[B29] Node K Inoue T Postprandial hyperglycemia as an etiological factor in vascular failure Cardiovasc Diabetol 2009 8 23 10.1186/1475-2840-8-23 19402896 PMC2688503

[B30] Knudsen EC Seljeflot I Michael A Eritsland J Mangschau A Müller C Arnesen H Andersen GØ Increased levels of CRP and MCP-1 are associated with previously unknown abnormal glucose regulation in patients with acute STEMI: a cohort study Cardiovasc Diabetol 2010 9 47 10.1186/1475-2840-9-47 20809989 PMC2940874

[B31] Wallander M Malmberg K Norhammar A Rydén L Tenerz A Oral glucose tolerance test: a reliable tool for early detection of glucose abnormalities in patients with acute myocardial infarction in clinical practice: a report on repeated oral glucose tolerance tests from the GAMI study Diabetes Care 2008 31 36 8 10.2337/dc07-1552 17909086

[B32] Bartnik M Rydén L Malmberg K Ohrvik J Pyörälä K Standl E Ferrari R Simoons M Soler-Soler J Euro Heart Survey Investigators Oral glucose tolerance test is needed for appropriate classification of glucose regulation in patients with coronary artery disease: a report from the Euro Heart Survey on Diabetes and the Heart Heart 2007 93 72 7 10.1136/hrt.2005.086975 16905628 PMC1861359

[B33] Bartnik M Ryden L Ferrari R Malmberg K Pyorala K Simoons M Standl E Soler-Soler J Ohrvik J The prevalence of abnormal glucose regulation in patients with coronary artery disease across Europe. The Euro Heart Survey on diabetes and the heart Eur Heart J 2004 25 1880 1890 10.1016/j.ehj.2004.07.027 15522466

[B34] Hu DY Pan CY Yu JM China Heart Survey Group The relationship between coronary artery disease and abnormal glucose regulation in China: the China Heart Survey Eur Heart J 2006 27 2573 9 10.1093/eurheartj/ehl207 16984927

[B35] Sourij H Saely CH Schmid F Zweiker R Marte T Wascher TC Drexel H Post-challenge hyperglycaemia is strongly associated with future macrovascular events and total mortality in angiographied coronary patients Eur Heart J 2010 31 1583 90 10.1093/eurheartj/ehq099 20436047

[B36] Feinberg MS Schwartz R Tanne D Fisman EZ Hod H Zahger D Schwammethal E Eldar M Behar S Tenenbaum A Impact of the metabolic syndrome on the clinical outcomes of non-clinically diagnosed diabetic patients with acute coronary syndrome Am J Cardiol 2007 99 667 72 10.1016/j.amjcard.2006.10.023 17317369

[B37] Fisman EZ Motro M Tenenbaum A Boyko V Mandelzweig L Behar S Impaired fasting glucose concentrations in nondiabetic patients with ischemic heart disease: a marker for a worse prognosis Am Heart J 2001 141 485 90 10.1067/mhj.2001.113219 11231448

[B38] Anselmino M Ohrvik J Malmberg K Standl E Ryden L Glucose lowering treatment in patients with coronary artery disease is prognostically important not only in established but also in newly detected diabetes mellitus: a report from the Euro Heart Survey on Diabetes and the Heart Eur Heart J 2008 29 177 184 10.1093/eurheartj/ehm519 18156611

[B39] Dormandy JA Charbonnel B Eckland DJ Erdmann E Massi-Benedetti M Moules IK Skene AM Tan MH Lefebvre PJ Murray GD Standl E Wilcox RG Wilhelmsen L Betteridge J Birkeland K Golay A Heine RJ Koranyi L Laakso M Mokan M Norkus A Pirags V Podar T Scheen A Scherbaum W Schernthaner G Schmitz O Skrha J Smith U Taton J Secondary prevention of macrovascular events in patients with type 2 diabetes in the PROactive Study (PROspective pioglitAzone Clinical Trial In macroVascular Events): a randomised controlled trial Lancet 2005 366 1279 1289 10.1016/S0140-6736(05)67528-9 16214598

[B40] Chiasson JL Josse RG Gomis R Hanefeld M Karasik A Laakso M Acarbose treatment and the risk of cardiovascular disease and hypertension in patients with impaired glucose tolerance: the STOP-NIDDM trial JAMA 2003 290 486 494 10.1001/jama.290.4.486 12876091

[B41] Kragelund C Snorgaard O Køber L Bengtsson B Ottesen M Højberg S Olesen C Kjaergaard JJ Carlsen J Torp-Petersen C TRACE Study Group Hyperinsulinaemia is associated with increased long-term mortality following acute myocardial infarction in non-diabetic patients Eur Heart J 2004 25 1891 7 10.1016/j.ehj.2004.07.033 15522467

